# Temporal Gap-Aware Attention Model for Temporal Action Proposal Generation

**DOI:** 10.3390/jimaging10120307

**Published:** 2024-11-29

**Authors:** Sorn Sooksatra, Sitapa Watcharapinchai

**Affiliations:** National Electronic and Computer Technology Center, National Science and Technology Development Agency, Khlong Nueng, Khlong Luang District, Pathum Thani 12120, Thailand; sorn.soo@nectec.or.th

**Keywords:** temporal action proposal generation, attention mechanism, contiguous action proposal

## Abstract

Temporal action proposal generation is a method for extracting temporal action instances or proposals from untrimmed videos. Existing methods often struggle to segment contiguous action proposals, which are a group of action boundaries with small temporal gaps. To address this limitation, we propose incorporating an attention mechanism to weigh the importance of each proposal within a contiguous group. This mechanism leverages the gap displacement between proposals to calculate attention scores, enabling a more accurate localization of action boundaries. We evaluate our method against a state-of-the-art boundary-based baseline on ActivityNet v1.3 and Thumos 2014 datasets. The experimental results demonstrate that our approach significantly improves the performance of short-duration and contiguous action proposals, achieving an average recall of 78.22%.

## 1. Introduction

A massive number of videos are now available from various sources, both online and offline. The content of these videos varies from entertainment and personal to educational and security content. These videos are generally long and untrimmed, containing both the background and key content. The key content is usually the motion or movement of humans or objects. Thus, action is one of the main key features in video analysis [1]. Extracting the key content from untrimmed videos is an important step for several applications. Video classification and action recognition usually require the video to be trimmed into a short clip for processing. Temporal action localization plays the main role in extracting and recognizing action instances or proposals. Temporal action localization also has extensive applications and value in the fields of video summarization [2], video surveillance [3], skill assessment [4], and everyday video recording. Despite significant progress and promising performance in action recognition, action instance extraction, called temporal action proposal generation (TAPG), still has a substantial need for improvement. The quality of the action proposal generation usually affects the performance of action localization and recognition. Hence, several recent studies have aimed to improve the TAPG performance in order to be utilized as input data for action localization and recognition.

**Figure 1:**
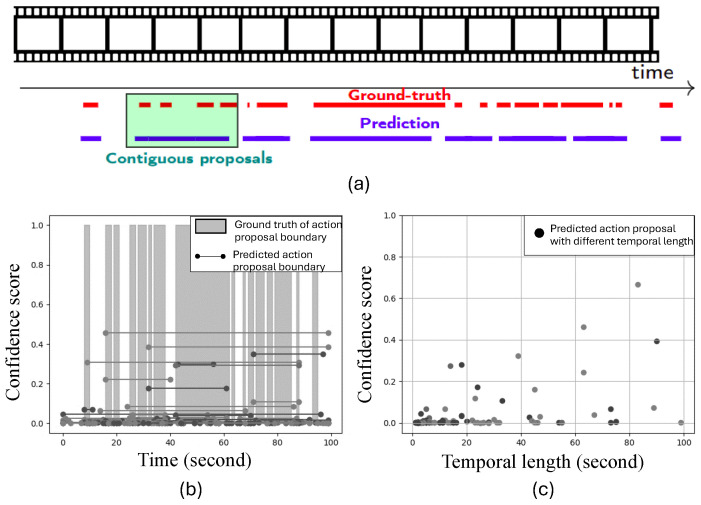
(**a**) An example of a video frame sequence with contiguous action proposals (within the green box) in predicted results, (**b**) the BMN candidate action proposal boundaries in different intensities with confidence scores, with actual proposals shown as gray shaded regions, and (**c**) the confidence scores on proposals by temporal length (best viewed in color).

TAPG is a technique for classifying whether the temporal proposals from untrimmed videos are background or action proposals. The background proposal represents the time periods of the video without key content. In most previous studies, various temporal lengths in action proposals have been one of the main issues in TAPG, especially in action proposals with short temporal lengths (<60 s). Similar to a multi-scale issue in object detection, TAPG should be capable of identifying action proposals of various temporal lengths. Previous studies have solved this issue by enriching various contexts in the temporal length and designing the objective function for imbalanced data for proposal extraction. As observed in the well-known TAPG model of the Boundary-Matching Network (BMN) [5], the cause of low prediction performance in small action proposals may be related to the merging of contiguous action proposals. Figure 1 shows an example of videos with contiguous action proposals.

Some contiguous proposals are merged in the prediction shown as a green box in Figure 1a. This merging problem is usually caused by low confidence scores for small action proposals. Figure 1b shows predicted proposal boundaries, represented as lines of different intensities with confidence scores, and the actual proposals represented, as gray-shaded regions along the time axis. Figure 1c shows that several predicted proposals with long temporal lengths have high confidence scores. However, the proposals merge several actual proposals with small temporal lengths and their temporal gaps. These results indicate that the traditional method should focus on temporal gaps to solve this merging problem.

This paper addresses the issue of merging contiguous action proposals by proposing a temporal gap-aware attention model. Inspired by the attention mechanism [6] in computer vision, attention masks are created to focus on target regions. The proposed method adapts the traditional TAPG method by generating an attention mask. The score within the attention mask is calculated based on the gap displacement between contiguous action proposals, especially those with a short temporal length. If the gap displacement between the two proposals is closer, the generated score between the two proposals in the attention mask will be higher. The hypothesis is that this TAPG model could achieve higher performance for small action proposals by emphasizing the regions of interest in the attention mask. The proposed model, called G-MCBD, uses Multi-Level Content-Aware Boundary Detection (MCBD) [7] as a baseline network and adds an attention module. The contributions of this paper are summarized as follows:

- We introduce G-MCBD, a gap-aware attention model for TAPG, designed to more effectively handle action proposals with various temporal lengths, especially those with a short temporal length.
- We propose an attention mechanism that generates a gap-aware attention mask to mitigate the merging problem in contiguous action proposals.

The rest of this paper is organized as follows: Section 2 provides a brief literature review of TAPG and attention mechanisms; Section 3 describes the preparation of gap-attention masks; Section 4 presents the overall G-MCBD architecture with its optimization and inference phases; empirical results are analyzed in Section 5; Section 6 provides result discussion related to practical application and final conclusions are presented in Section 7.

## 2. Related Work

This section briefly describes the contributions of related studies in temporal action proposal generation, focusing on deep-learning-based techniques. Relevant literature on attention mechanisms is also included in this section.

### 2.1. Temporal Action Proposal Generation

There has been a considerable amount of research on TAPG in recent years. In general, TAPG is categorized into the anchor-based and boundary-based methods. The anchor-based method, adapted from object detection, uses sliding windows or pre-defined temporal anchors [8,9]. This method requires a large computational time to extract both spatial and temporal features. A self-adaptive proposal [10] has been proposed to randomly select salient temporal proposals. In addition, the starting and ending periods of the action proposal, other stages (e.g., ready, conform, etc.) are included in a multi-stage network [11]. Even though the anchor-based method is able to enrich temporal features from action proposals, it is unable to adapt to various temporal lengths and has imprecise proposal boundaries due to the fixed length of candidate proposals. The boundary-based method has recently been introduced to solve this problem. Boundary prediction uses frame-level context information around the proposal boundaries [5,7,12,13,14]. These methods focus on evaluating confidence scores and boundary probabilities for each temporal proposal. Since the boundary-based method is sensitive to noise, rich confidence scores cannot be extracted from each candidate compared to the anchor-based method. Therefore, additional techniques are needed to help extract proposal features (e.g., U-shape architecture [11], graph neural network [15], bi-directional encoder [16]). These techniques are able to capture temporal features for action proposals of various temporal lengths. Ref. [17] proposed integrating an inter-instance contrastive learning mechanism to play a regularizing role in boundary prediction. In addition, ref. [18] proposed SMBG to reduce time complexity in boundary-based methods.

### 2.2. Attention Mechanism

Since valuable features are difficult to extract from complex visual scenes in an image or video, the attention mechanism can help the model focus on target or salient regions. This module is generally implemented at multiple levels of the classification network by formulating the attention mask as a region of interest. In object detection, Faster R-CNN [19] is used as a unit in the convolutional neural network (CNN) model to reduce overfitting errors. The attention model is applied to convolutional feature maps for both channel-wise [20] and spatial [6,21] domains. Attention mechanisms are also gaining attention for extracting action proposals from the temporal domain. Because people are generally the main agent performing any actions or motions, the agent-environment network (AEN) [22] focuses on candidate proposals containing people. Objects related to human action are included as proposal features in [23]. The background proposal suppression technique is also used in the network of [24] to suppress temporal features from background proposals. Furthermore, the self-attention of the vision transformer [9] can be adapted to extract the most salient feature in the temporal domain.

The boundary-based TAPG methods that have been used in recent years, and their contributions, are summarized in Table 1. Most recently, studies on TAPG have focused on the attention mechanism to improve the performance of action proposal extraction. This line of research has focused on various purposes of AEN and AOE-Net, such as handling various temporal lengths or extracting salient features. However, the subpar performance that results from the merging of contiguous action proposals is not addressed in existing TAPG studies. Therefore, a gap-aware attention module is proposed in this paper to overcome this limitation and improve the performance of the TAPG model.

## 3. Data Preparation

### 3.1. Problem Definition

Untrimmed video (*X*) can be denoted as the sequence of frames for any temporal length containing *L* frames. This sequence is expressed as *X*=${\left\{x_{n}\right\}}_{n=1}^{L}$, where $x_{n}$ is the $n^{th}$ frame in the input video. The video descriptor of an input video (*F*) is divided with a specific period (*T* = ⌊$\frac{L}{\delta }$⌋, where δ is the number of non-overlapped frame snippets), using the following formula:

(1) $$F={\left\{f_{i}\right\}}_{i=1}^{T}={\left\{\phi \left(x_{\delta \cdot (i-1)+1},...,x_{\delta \cdot i}\right)\right\}}_{i=1}^{T}$$

where ϕ is a function for extracting the video descriptor. Since a boundary-based method was used in this paper, the general data annotation was divided into $g_{s}$, $g_{e}$, and $g_{p}$ as the probability of starting time, ending time, and confidence map, respectively. In this paper, the preparation process was carried out according to the methods used in BMN [5].

### 3.2. Gap-Aware Attention Mask

This paper focused on preventing contiguous action proposals from merging. The gap-aware attention mask was proposed via the attention mechanism. Our attention mask was prepared using a temporal boundary consisting of starting time ($g_{s}$) and ending time ($g_{e}$) to emphasize the gap between proposals. Then, all action proposals were arranged as ([$g_{s}^{n}$, $g_{e}^{n}$], [$g_{s}^{\left(n+1\right)}$, $g_{e}^{\left(n+1\right)}$], …, [$g_{s}^{N}$, $g_{e}^{N}$]) ordered by starting time, where *N* is the number of action proposals. The score in the attention mask was inspired by the probability density function between neighbor proposals. All temporal center positions between two proposals or within the temporal gap were calculated ($\mu_{n}=\frac{g_{s}^{n+1}-g_{e}^{n}}{2}$). Then, the rest temporal positions were utilized to calculate a normal distribution and an attention score as using the following formula:

(2) $$S_{n}=max{\left[\frac{1}{\sigma \sqrt{2\pi }}e^{-\frac{y_{i}-\mu_{i}}{2\sigma^{2}}}\right]}_{i=n}^{n+1}$$

where $S_{n}$ and $\mu_{n}$ are scores in the attention mask and the temporal center position, respectively, $y_{n}$ represents a temporal position between $g_{e}^{n}$ and $g_{s}^{n+1}$ in a 2D confidence map, and standard deviation is represented as σ.The score is a maximum value of normal distribution and depends on the displacement between action proposals. Smaller displacement generates a higher score. Cumulative summation is the gap-aware attention mask ($G_{a}$) of all scores, as shown in Equation (3).

(3) $$G_{a}=\sum_{n=0}^{N-1}S_{n}$$

**Figure 2:**
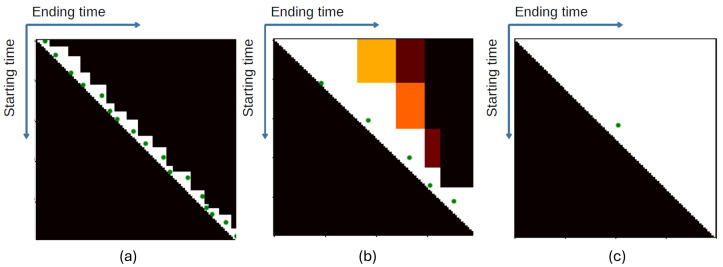
Examples of attention masks from videos with various gap displacements in the range of (**a**) 0–10 s, (**b**) 20–30 s, and (**c**) more than 100 s, where green dots represent the action proposal positions. (Best viewed in color.)

Figure 2 shows an example of attention masks from three videos with different ranges of gap displacement based on the number of action proposals. The green circles indicate the positions of the action proposals in each video. Attention masks emphasized the area around the action proposals. The attention masks shown in Figure 2a of a video with a small gap displacement range indicate that higher scores were obtained around the contiguous action proposals. Conversely, scores of long action proposals (upper right corner) were suppressed by this technique. With a larger range of gap displacements, as shown in Figure 2b, the emphasized region was enlarged. Nevertheless, this attention mask was ineffective for cases with a small number of action proposals, such as in Figure 2c, where all regions in the attention map were equally emphasized.

### 3.3. Label Assignment

Following BMN [5] and BSN [13], the ground-truth for network optimization included starting labels ($G_{s}$), ending labels ($G_{e}$), and confidence-map labels ($G_{c}$). The temporal labels (starting and ending time) were generated in every snippet of input videos and were calculated using the following formula:

(4) $$G_{s}={\left\{g_{s}^{n}\right\}}_{n=0}^{T},G_{e}={\left\{g_{e}^{n}\right\}}_{n=0}^{T}$$

where the time position at each $g_{s}^{n}$ and $g_{e}^{n}$ was set to 1 if its corresponding timestamp in the video was the nearest to any ground-truth starting or ending timestamp. Confidence-map labels for each video had dimensions (D×T) and ranged from 0 to 1, where *D* represents the maximum length of proposals. In our experiment, we set D=T. The score in every temporal position in $G_{c}$ was calculated using the intersection of union (IoU) compared with action proposal positions.

## 4. Overview of G-MCBD Architecture

**Figure 3:**
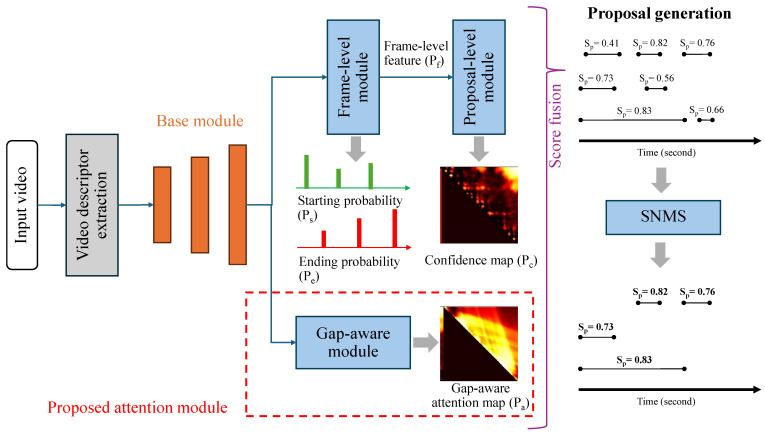
The proposed TAPG network architecture (G-MCBD) with score fusion and SNMS in an inference phase. Green and red circles represent the starting and ending time of each action proposal, respectively (best viewed in color).

Our proposed attention module can serve as an extension to a boundary-based method in an end-to-end framework, as shown in Figure 3. In this paper, a recent boundary-based method called MCBD [7] is modified to be used as the main backbone network because of its promising and impressive performance. MCBD architecture contains three main modules: a base module (BM), a frame-level module (FM), and a proposal-level module (PM). The detailed architecture of each module is summarized in Table 2. The first module, BM, processes a video descriptor with the stack of convolutional layers and then feeds the video features to the other modules. The action proposal boundary is the main target in FM for producing probabilities of the starting ($P_{s}$) and ending time ($P_{e}$) in each temporal position and extracting frame-level features ($P_{f}$) from an intermediate layer (Table 2). Lastly, PM helps produce a 2D metric of confidence scores for every possible temporal position. The PM ignores the regions where the ending time is earlier than the starting time in the confidence maps ($P_{c}$), as shown in Figure 3. In addition, this paper proposes an attention module called the gap-aware module (GM), also shown in Figure 3. This module was used to generate the gap-aware attention mask to emphasize contiguous action proposals, described in the next subsection.

### 4.1. Gap-Aware Module

The main contribution of this paper is the attention mechanism using gap-aware attention masks. Instead of generating 2D confidence maps, the proposed model generates 2D maps based on the gap displacement between the proposals. The GM architecture, as shown in Table 2, is based on PM with the boundary-matching layer transforming 1D temporal features into 2D feature maps for feeding into the stack of 2D convolutional layers. The number of sample points (N) was set to 32, and the maximum duration (D) of the proposal depended on the size of the dataset. The predicted result of this module ($P_{a}$) was optimized using temporal annotation.

### 4.2. Network Optimization

This section describes the details of the training process and optimization of the model parameter. Our network aims to optimize weights in three modules (FM, PM, and GM). Our TAPG network consists of five outputs from the three modules: the probability vectors of starting ($P_{s}$) and ending time ($P_{e}$) from FM, the 2D confidence maps ($P_{c}^{b}$ and $P_{c}^{r}$) from PM, and a gap-aware attention mask ($P_{a}$) from GM. Following [5,7], these confidence maps were trained using binary and regression losses ($P_{c}^{b}$ and $P_{c}^{r}$). The losses from FM, PM, and GM, represented by $L_{FM}$, $L_{PM}$, and $L_{GM}$, respectively, were optimized simultaneously according to Equations (5)–(8):

(5) $$L_{FM}=L_{bl}\left(P_{s},G_{s}\right)+L_{bl}\left(P_{e},G_{e}\right)$$

(6) $$L_{PM}=L_{bl}\left(P_{c}^{b},G_{c}\right)+\lambda_{r}L_{2}\left(P_{c}^{r},G_{c}\right)$$

(7) $$L_{GM}=\lambda_{r}L_{2}\left(P_{a},G_{a}\right)$$

(8) $$L=\lambda_{1}L_{FM}+\lambda_{2}L_{PM}+\lambda_{3}L_{GM}$$

where *L* is the overall loss for our TAPG network. Following [7], $\lambda_{r}$ was set to 10, and $\lambda_{1},\lambda_{2},$ and $\lambda_{3}$ were set to 1. The least square error is expressed as $L_{2}$. $L_{bl}$ is a weighted binary log-likelihood function to solve the imbalanced number between action and background proposals. This loss function was calculated using Equation (9)

(9) $$L_{bl}=\frac{\sum_{i=1}^{N}\left[\frac{N\cdot g_{i}\cdot log\left(p_{i}\right)}{N_{p}}+\frac{N\cdot \left(1-g_{i}\right)\cdot log\left(p_{i}\right)}{N_{n}}\right]}{N}$$

where $g_{i}$ and $p_{i}$ are ground truths and predicted results, respectively, and the number of all proposals, action proposals, and background proposals are represented as *N*, $N_{p}$, and $N_{n}$, respectively.

### 4.3. Proposal Generation

In the inference or testing phase, the predicted action proposals were generated by analyzing the outputs from the three modules. With $t_{s}$ and $t_{e}$ indicating the temporal indices for starting and ending times, respectively, the score of the predicted action proposals was calculated using Equation (10), based on [5]:

(10) $$S_{p}=P_{s}\left[t_{s}\right]P_{e}\left[t_{e}\right]\sqrt{P_{c}^{b}\left[d_{p},t_{s}\right]P_{c}^{r}\left[d_{p},t_{s}\right]}P_{a}\left[d_{p},t_{s}\right]$$

where $S_{p}$ is the action proposal score. $d_{p}$ is the duration between $t_{s}$ and $t_{e}$. Then, these scores were ordered using soft non-maximum suppression (SNMS) [25] to eliminate highly overlapped proposals, as shown in Figure 3. The final output is a list of selected action proposals.

## 5. Experiments

### 5.1. Experimental Setup

Dataset: TAPG evaluation in this experiment was performed using ActivityNet v1.3 [26] and Thumos 2014 [27]. The first dataset comprised 19,994 videos from YouTube with an average video length of 115 s per video. Videos were divided into training, testing, and validation sets with a ratio of 2:1:1, respectively. The exact number of videos used for training, validation, and testing was 10,024, 4926, and 5044 videos, respectively. For action instances, the average number of actions in each video was about 1.54, and the total number of actions was 200 and 214 in the training and validation sets, respectively. There were 20 action categories. The average video length was 7.21 s per video with an average temporal length of 4.6 s for the proposals. Unlike videos in ActivityNet v1.3, each video from Thusmos 2014 may contain two or more action classes, leading to the overlapping of several action proposals. Following [28], the action proposals were categorized based on their duration or temporal length as follows:

- Extra short (XS): temporal length from 0 to 30 s.
- Short (S): temporal length from 30 to 60 s.
- Medium (M): temporal length from 60 to 120 s.
- Long (L): temporal length from 120 to 180 s.
- Extra long (XL): temporal length of more than 180.

Implementation detail: For the all-video dataset, the TSP network [28] was used as a video descriptor with a feature length of =512. The specific time period (*T*) was set as 100 with the number of non-overlapped frame snippets set to 16 frames. Following [7], the proposed network (G-MCBD) was optimized using the Adam optimizer with a learning rate of $10^{-5}$ from the training set. The model evaluation was performed using the validation set.

### 5.2. Evaluation Metric

This experiment followed the standard criteria for TAPG evaluation using average recall (AR), where IoU was used as the threshold. The AR in ActivityNet v1.3 was [0.5:0.05:0.95], and the AR in Thumos 2014 was [0.5:0.05:1.00]. This criterion assesses the accuracy of finding correct action proposals. In general, the TAPG network should be tested on AR with several sets of average number of proposals (AN), which is documented as AR@AN. In ActivityNet v1.3, AR@100 (AN = 100) is the primary average recall for evaluation. Conversely, Thumos 2014 evaluates AR with five AN values: AR@50, AR@100, AR@200, AR@500, and AR@1000. In addition, to analyze network stability using AN, a mean value of AR from AN = 0 to 100 was calculated from the area under the curve (AUC) between AR and AN, in ActivityNet v1.3.

### 5.3. Ablation Study

In this paper, our goal is to solve the problem of a contiguous proposal by proposing a gap-aware attention module. This attention module can be included in TAPG network to improve performance, especially in a contiguous proposal. In an ablation study, the backbone network (MCBD), without a gap-aware attention module, was implemented to be compared with G-MCBD. Their performance was evaluated from the following perspectives:

#### 5.3.1. Effect on Gap Displacement

Gap displacement might be a problem when detecting small action proposals. There are currently no standard criteria for categorizing action proposals with different gap displacements. In this experiment, the gap displacement of action proposals was calculated using the boundary distance from their shortest neighbor action proposals. Our observations indicated that most contiguous action proposals had gap displacements of less than 2 min, so action proposals with large displacements were excluded from this analysis. Action proposals were categorized into four groups based on gap displacements in seconds (0–10, 10–20, 20–30, and 30–100), as shown in Table 3 and Table 4 for ActivityNet v1.3 and Thumos 2014, respectively. Higher values of AR@100 and AUC indicated a lower chance of merging proposals. The empirical results from ActivityNet v1.3 revealed that more action proposals could be extracted using G-MCBD, which was especially effective in videos with small gap displacements. However, the proposed method was ineffective in Thumos 2014. As the AN increased, the proposed method insignificantly improved from the baseline MCBD.

#### 5.3.2. Effect on Temporal Length

According to the methods outlined by [28], action proposals were categorized into five groups, based on their temporal length in seconds (0–30, 30–60, 60–120, 120–180, >180) to evaluate each method’s performance using the length of action proposals. The empirical results are shown in Table 5 and Table 6 for ActivityNet v1.3 and Thumos 2014, respectively. Table 5 shows that the proposed method, G-MCBD, was effective on action proposals with small temporal lengths, especially those under 30 s. However, on action proposals with large temporal lengths, G-MCBD had a lower AR@100 and AUC than its baseline network because their confidence scores were reduced from gap-aware attention masks. The overall performance of G-MCBD was slightly better than the other two methods, especially in S and M. Unfortunately, the action proposals from Thumos 2014 only had small temporal lengths (XS, S, and M), so only the results from those three temporal-length ranges are shown in Table 6. Similar to the results in ActivityNet v1.3, the proposed method outperformed other methods for action proposals with short temporal lengths in Thumos 2014. However, the performance decreased with a lower AN, resulting in a lower number of enriched action proposals.

#### 5.3.3. Effect on Overlapping Action Proposals

Given the characteristics of Thumos 2014, there were several overlapping action proposals with different action classes. As observed in Thumos 2014, the number of videos with overlapping action proposals accounted for 15.49% of the dataset. Table 7 categorizes the experimental results into videos with overlapping action proposals and those with non-overlapping proposals. The result clearly showed that the AR from non-overlapping proposals was higher than the AR from overlapping proposals for all methods. Gap-aware attention masks were effective for videos with non-overlapping proposals, outperforming the other two methods. However, their performance was generally lower in videos with overlapping proposals. Since the gap-aware attention mask was designed to focus on the gap between proposals of the same class, overlapping proposals might be ignored, decreasing TAPG performance.

#### 5.3.4. Effect on Computational Complexity

Computational complexity is an important factor in model evaluation. Computational complexity was compared between G-MCBD and its baseline network, MCBD. AOE-Net was also included in the comparison as a recent TAPG with an attention mechanism. The experiment was tested on an NVIDIA Tesla K20m. Computational complexity was divided into two parts, consisting of the time for model inference ($T_{inf}$) and the time for proposal generation ($T_{pro}$), as illustrated in Table 8. The results clearly showed that the attention mechanism only affected $T_{inf}$, while all methods had equivalent computational complexity for proposal generation. Even though the proposed method had a higher $T_{inf}$, around 2.5× that of MCBD, G-MCBD was faster than AOE-Net, which had a $T_{inf}$ around 2.7× that of MCBD.

### 5.4. Comparison with State-of-the-Art Methods

This section describes the experimental results of a state-of-the-art method (SOTA) performance comparison and an ablation study of the proposed network (G-MCBD). SOTAs were gathered from studies from the last few years related to boundary-based methods and attention mechanisms. For a fair comparison, other methods using the same baseline network as the proposed method were also conducted to demonstrate the contribution of the proposed method. The MCBD was implemented from a public source [29]. The attention model from AOE-Net was obtained from [30].

The validation sets in ActivityNet v1.3 and Thumos 2014 were the primary focus of this comparison. In ActivityNet v1.3, AR@100 and AUC were obtained from the methods of each publication, as shown in Table 9. The results showed that MCBD had the best performance in both AR@100 and AUC compared with other SOTAs. However, the proposed method underperformed compared to its baseline network (MCBD), showing similar performance in AR@100 and AUC. This indicates that including a gap-aware module for MCBD resulted in an insignificant improvement in overall performance.

Table 10 shows the overall performance of Thumos 2014 compared with other SOTAs. The results clearly show that MCBD achieved the best performance with a small AN (50 and 100). However, the proposed method was able to achieve better performance than the baseline MCBD with higher AN values (200, 500, and 1000). Compared to ActivityNet v1.3, the data in Thumos 2014 contains overlapping proposals, which might increase difficulty and lead to lower performance.

### 5.5. Qualitative Comparisons

**Figure 4:**
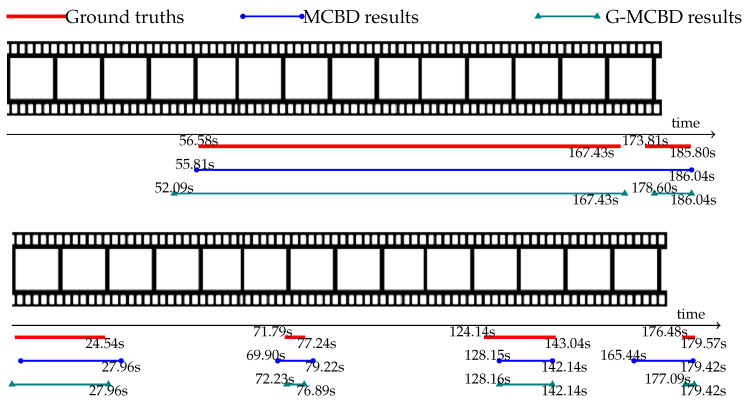
Examples of successful cases in merging contiguous proposals (**top**) and emphasizing small action proposals (**bottom**) with predicted proposals from MCBD (blue lines), G-MCBD (green lines), and their ground truths (red lines). The predicted starting and ending times of each are indicated by the beginning and ending of each line, respectively (best viewed in color).

**Figure 5:**
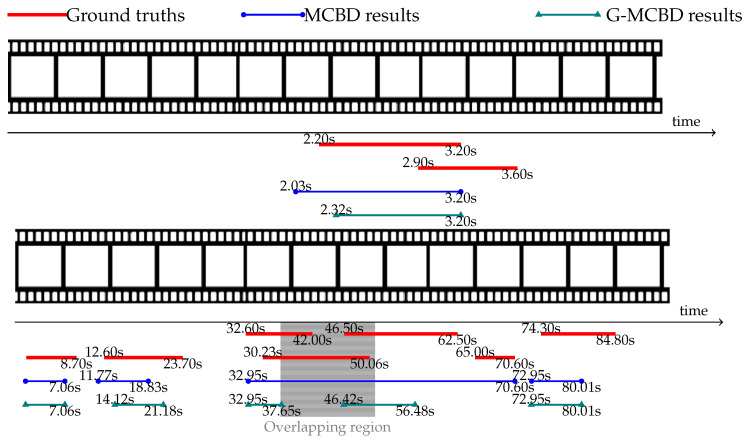
Examples of failure cases in overlapping proposals (**top**) and proposals within temporal gaps (**bottom**) with predicted proposals from MCBD (blue lines), G-MCBD (green lines), and their ground truths (red lines). The predicted starting and ending times of each are indicated by the beginning and ending of each line, respectively (best viewed in color).

Qualitative results are visualized in Figure 4 and Figure 5, showing predicted proposals from MCBD (blue lines) and G-MCBD (green lines) compared with their ground truths (red lines). The top image of Figure 4 shows an example of the predicted result from MCBD where two contiguous proposals were merged into a single blue line. In contrast, the proposed method successfully separated the two contiguous proposals into two distinct green lines. In small action proposals, the proposed method was able to localize the short proposal more precisely, as shown in the bottom image of Figure 4.

Figure 5 illustrates the example of overlapping action proposals of different classes from the Thumos 2014 dataset. The red lines in different levels represent the action proposals of different action classes. Given that G-MCBD and MCBD were optimized to localize only the action proposal, these methods could not differentiate between proposals of different action classes. Therefore, the merging problem also affected overlapping proposals, as shown in the top image of Figure 5. The bottom image of Figure 5 shows an example of a proposal located within the temporal gap of an action proposal from another action class. In G-MCBD, the gap-attention mask was designed to emphasize the temporal gap and remove any action proposals overlapping with these temporal gaps. Because of this, the proposal within these temporal gaps was ignored by G-MCBD, as shown in the gray highlighted region of Figure 5.

## 6. Discussion

Actions can vary significantly in their temporal lengths, ranging from short-duration actions, such as head nodding, to longer-duration actions, like putting on clothes. Short-duration actions are particularly challenging to detect due to their rapid execution. Contiguous proposals, on the other hand, refer to separate actions that occur in close temporal proximity, often with small gaps between them, making it more difficult to separate these proposals. The experimental results indicated that the proposed techniques were effective in extracting proposals with small temporal lengths and accurately detecting action instances in contiguous proposals. The accurate detection of actions with short duration or within contiguous proposals has significant implications for various real-world applications, as follows:

- Video search and retrieval: improving search accuracy by allowing users to query for specific, short-duration actions.
- Human–computer interaction: enabling more intuitive interaction with a sequence of actions and a small gap displacement.
- Video summarization: creating more concise and informative features from short-duration action segments and contiguous proposals.
- Video surveillance: facilitating the detection of suspicious or anomalous behavior that usually occurs for a short duration.

While our proposed technique has shown significant improvement and promising results, there are still limitations to be addressed, as follows:

- Focusing on the temporal gap in action proposals could allow action proposals with large proposal lengths to be misclassified as a group of short actions in contiguous proposals, resulting in lower performance for a long-action instance.
- Our gap-aware attention module was designed to suppress any action instance within the temporal gap region. Accordingly, this method could not handle action proposals that overlap with these temporal gaps.

## 7. Conclusions

In this paper, we have proposed a gap-aware attention module for the TAPG model. The proposed method was designed to handle action proposals of various temporal lengths and to prevent the merging of proposals with small gap displacements. Based on our observation, the merging of contiguous action proposals was a contributing factor to poor performance in extracting small action proposals. MCBD, a boundary-based method, was used as the baseline model of the proposed method, called G-MCBD, which adds a gap-aware attention module. This attention module was designed using the structure of proposal modules to generate attention masks. The scores from these attention masks were based on the gap displacement of action proposals to emphasize contiguous and small action proposals. In the ablation study, MCBD and AOE-Net were used for comparison to evaluate the performance of G-MCBD. The empirical results revealed the effectiveness of the proposed method, particularly on action proposals with small temporal lengths or with small gap displacements. In addition, the action instances within contiguous were successfully separated. Analyzing the results revealed that the proposed method could be applied in video analysis applications, enabling more detailed analysis of short action and continuous action sequences.

While the proposed method shows promising potential, there is still room for improvement in terms of network optimization and inner architecture. In future work, we plan to explore other baseline networks and datasets for TAPG networks. Multi-attention masks will be designed for multiple action classes to solve overlapping action proposals in Thumos 2014. In addition, a further analysis of practical applications will be discussed to find a significant limitation.

## Figures and Tables

**Table 1:** The contributions of different boundary-based TAPG methods.

Methods	Contribution
BSN [13]	- Proposing Boundary-Sensitive Network (BSN) - Designing temporal and proposal modules to handle the ‘local to global’ fashion for boundary and proposal prediction, respectively.
BMN [5]	- Proposing Boundary-Matching Network (BMN) - Jointly unifying framework between temporal relation and proposal generation modules.
DBG [14]	- Proposing Dense Boundary Generator (DBG) - Redesigning information from BMN and BSN as 2D information between starting and ending times. - Introducing auxiliary supervision via confidence-score classification.
BSN++ [16]	- Exploiting a new framework of BSN (BSN++). - Introducing a mechanism to evaluate densely distributed proposals from their confidence scores.
RTD-Net [9]	- Presenting Relaxed Transformer Decoder Network (RTD-Net). - Adapting the transformer for object detection to visualize the global view of an action proposal and reduce time complexity. - Generating proposals via a relaxation mechanism to reduce ambiguous characteristics between action and background proposals.
AEN [22]	- Proposing Agent-Environment Network (AEN). - Fusing human and environment features interacting with action into boundary-based methods as agent and environment pathways, respectively.
AOE-Net [23]	- Proposing Actors-Objects-Environment Interaction Network (AOE-Net) - Extracting semantic relations between main actors and relevant objects while ignoring inessential actors in scenes.
MCBD [7]	- Introducing Multi-Level Content-Aware Boundary Detection (MCBD). - Using complementary information from proposal boundaries and content to overcome action proposals with indeterminate boundaries. - Formulating multi-level information in temporal action proposal generation in multi-dimensional forms.

**Table 2:** The detailed architecture of the proposed TAPG network, where the BM layer is the boundary-matching layer.

Layer List	Kernel	Dimension	Output Size
Base Module (BM)			
1D Conv	3	256	256×T
1D Conv	3	128	128×T
Frame-level module (FM)			
1D Conv	3	256	256×T
1D Conv	3	128	$P_{f}:128\times T$
1D Conv	3	2	$\{P_{s},P_{e}\}:2\times T$
Proposal-level module (PM)			
BM_layer	N = 32		128×32×D×T
3D Conv	32×1×1	512	512×1×D×T
Squeeze			512×D×T
2D Conv	1×1	128	128×D×T
2D Conv	3×3	128	128×D×T
2D Conv	1×1	2	$\{P_{c}^{b},P_{e}^{r}\}:2\times D\times T$
Our gap-aware module (GM)			
BM_layer	N = 32		128×32×D×T
3D Conv	32×1×1	512	512×1×D×T
Squeeze			512×D×T
2D Conv	1×1	128	128×D×T
2D Conv	3×3	128	128×D×T
2D Conv	1×1	1	$P_{a}:1\times D\times T$

**Table 3:** The experimental results of MCBD and G-MCBD in action proposal generation with different gap displacements (in seconds) in ActivityNet v1.3.

Gap Displacement (Seconds)	AR@100 (%)	
	**MCBD**	**G-MCBD**
(0–10)	31.33	33.56
(10–20)	32.03	33.03
(20–30)	23.86	24.10
(30–100)	27.92	27.11
	AUC (%)	
(0–10)	32.00	32.24
(10–20)	31.03	31.98
(20–30)	23.53	23.77
(30–100)	28.42	28.09

**Table 4:** The experimental results of MCBD and G-MCBD in action proposal generation with different gap displacements (in seconds) in Thumos 2014.

Gap Displacement (Seconds)	AR@50 (%)	
	**MCBD**	**G-MCBD**
(0–10)	35.69	36.39
(10–20)	37.24	37.14
(20–30)	39.18	40.78
(30–100)	34.24	35.89
	AR@100 (%)	
(0–10)	42.66	42.58
(10–20)	45.99	45.56
(20–30)	46.10	46.95
(30–100)	43.47	41.83
	AR@200 (%)	
(0–10)	51.17	50.21
(10–20)	52.48	51.89
(20–30)	52.47	50.43
(30–100)	50.17	47.18
	AR@500 (%)	
(0–10)	56.87	56.82
(10–20)	59.24	58.93
(20–30)	57.65	57.11
(30–100)	53.47	53.18
	AR@1000 (%)	
(0–10)	59.11	59.18
(10–20)	60.50	60.54
(20–30)	59.14	59.23
(30–100)	54.45	54.37

**Table 5:** The experimental result of MCBD and G-MCBD performance in action proposal generation with action proposals of different temporal lengths (in seconds) in ActivityNet v1.3.

Temporal Length of Action Proposals (Seconds)	AR@100 (%)	
	**MCBD**	**G-MCBD**
XS (0–30)	50.03	53.19
S (30–60)	80.93	80.99
M (60–120)	90.24	90.24
L (120–180)	94.36	94.17
XL (>180)	95.88	95.98
	AUC (%)	
XS (0–30)	48.22	48.95
S (30–60)	77.76	77.83
M (60–120)	87.82	87.87
L (120–180)	92.93	92.76
XL (>180)	94.82	94.93

**Table 6:** The experimental result of MCBD, AOE-Net, and G-MCBD performance in action proposal generation with action proposals of different temporal lengths (in seconds) in Thumos 2014.

Temporal Length of Action Proposals (Seconds)	AR@50 (%)	
	**MCBD**	**G-MCBD**
XS (0–30)	38.41	39.21
S (30–60)	50.30	53.71
M (60–120)	79.09	80.45
	AR@100 (%)	
XS (0–30)	46.75	46.29
S (30–60)	53.56	58.48
M (60–120)	80.45	80.45
	AR@200 (%)	
XS (0–30)	55.32	53.78
S (30–60)	65.83	71.89
M (60–120)	80.45	80.45
	AR@500 (%)	
XS (0–30)	61.64	61.36
S (30–60)	79.69	76.66
M (60–120)	89.54	94.09
	AR@1000 (%)	
XS (0–30)	64.02	64.06
S (30–60)	83.10	92.95
M (60–120)	89.54	94.09

**Table 7:** The experimental result of MCBD and G-MCBD performance in action proposal generation in videos with non-overlapping and overlapping action proposals in Thumos 2014.

Non-Overlapping or Overlapping Action Proposals	AR@50 (%)	
	**MCBD**	**G-MCBD**
Non-overlap	39.07	39.89
Overlapping	36.01	37.24
	AR@100 (%)	
Non-overlap	47.04	47.30
Overlapping	44.25	43.02
	AR@200 (%)	
Non-overlap	55.55	54.26
Overlapping	55.11	53.09
	AR@500 (%)	
Non-overlap	61.00	61.95
Overlapping	61.41	61.07
	AR@1000 (%)	
Non-overlap	64.79	64.95
Overlapping	62.35	62.34

**Table 8:** The average computational complexity of MCBD, AOE-Net, and G-MCBD, as measured by the time for model inference and time for proposal generation.

Methods	$T_{\inf}$ (ms)	$T_{\mathrm{pro}}$ (ms)
MCBD	119.27	16.04
AOE-Net	313.62	19.12
G-MCBD	297.18	20.06

**Table 9:** SOTA methods comparison of overall TAPG performance on validation set of ActivityNet v1.3.

Methods	AR@100 (%)	AUC (%)
MGG [11]	74.52	66.43
BMN [5]	75.01	67.10
DBG [14]	76.65	68.23
BC-GNN [15]	76.73	68.05
BSN++ [16]	76.52	68.26
AEN [22]	75.65	68.15
RTD-Net [9]	73.21	65.78
AOE-Net [23]	76.43	68.42
MCBD [7]	78.29	69.90
G-MCBD	78.22	69.36

**Table 10:** SOTA comparison of overall TAPG performance on testing set of Thumos 2014.

Methods	AR@50 (%)	AR@100 (%)	AR@200 (%)	AR@500 (%)	AR@1000 (%)
MGG [11]	39.93	47.75	54.65	61.36	64.06
BMN [5]	40.69	47.99	55.76	62.18	64.48
DBG [14]	37.32	46.67	46.59	62.21	66.40
BC-GNN [15]	40.50	49.60	56.33	62.80	66.57
BSN++ [16]	42.44	49.84	57.61	65.17	66.83
AEN [22]	33.36	42.93	50.34	59.10	64.03
RTD-Net [9]	41.52	49.32	56.41	62.91	-
AOE-Net [23]	40.87	49.09	56.24	64.53	67.29
MCBD [7]	44.72	51.74	58.39	66.29	69.37
G-MCBD	44.50	51.65	59.05	66.77	69.53

## Data Availability

The ActivityNet v1.3 dataset is openly available at http://www.activity-net.org/ accessed on 2 August 2021, reference number [26], and the THUMOS 2014 dataset is openly available at http://crcv.ucf.edu/THUMOS14/download.html accessed on 21 April 2022, reference number [27].
